# La ética en la investigación social, sus ausencias, urgencias y posibilidades: propuestas críticas desde América Latina

**DOI:** 10.18294/sc.2025.5759

**Published:** 2025-08-08

**Authors:** Rubén Muñoz Martínez

**Affiliations:** 1 Doctor en Antropología Social. Investigador titular, Centro de Investigaciones y Estudios Superiores en Antropología Social (CIESAS), Ciudad de México, México. rubmuma@hotmail.com Centro de Investigaciones y Estudios Superiores en Antropología Social Centro de Investigaciones y Estudios Superiores en Antropología Social Ciudad de México Mexico rubmuma@hotmail.com

**Keywords:** Ética en Investigación, Comités de Ética en Investigación, Ciencias Sociales

## Abstract

La ética en la investigación social cualitativa tiene particularidades que la diferencian de la formulada desde el Norte Global para las ciencias de la salud, caracterizada por su dimensión universalista, cuantitativa, clínica y poco crítica respecto a su papel en las relaciones de poder. En la antropología social latinoamericana, y de manera similar a otras ciencias sociales, son escasas las discusiones colectivas institucionalizadas sobre cómo se está entendiendo, excluyendo y practicando en sus procesos investigativos. En este ensayo examino estas problemáticas en América Latina, así como algunas respuestas colegiadas en la academia y, desde la participación social, en las comunidades con las que trabajamos. Para ello analizo el riesgo/beneficio, el valor social, el consentimiento informado, la confidencialidad, la validez de los resultados, la ética en la investigación propuesta por dichas comunidades, para protegerse de los investigadores, y dos experiencias institucionales en las que colaboré, planteando preguntas y posibilidades con ejemplos en el campo de la antropología y la salud.

## INTRODUCCIÓN

El objetivo de este artículo es plantear algunas preguntas y discusiones respecto a la ética de la investigación social de tipo cualitativo, con ejemplos que refieren a campos disciplinarios y temáticos como el de la antropología y la salud. Su escritura surge a partir de lo que considero como ausencias, demandas y respuestas en diversos circuitos profesionales de las ciencias sociales en América Latina, al definirla y abordarla. Tres son los ejes de análisis del presente texto. El primero, refiere a la escasa discusión colectiva institucionalizada respecto a la ética en la investigación social cualitativa y sus repercusiones negativas en nuestra práctica profesional, teniendo en cuenta las limitaciones de una deliberación individual o de los acuerdos tácitos en las comunidades desde las que se lleva a cabo. El segundo, se relaciona con la potencial imposición de la hegemonía de las regulaciones formuladas desde el campo biomédico (en el Norte Global) que, a partir de una perspectiva universalista^1^^,^^2^, apegada al procedimentalismo y al principialismo^3^ y dominantemente cuantitativa-experimental-clínica, no contemplan -o lo hacen de forma restringida- las especificidades de otros tipos de investigaciones ni la dimensión política de la ética respecto a su papel en las relaciones de poder. Y, el tercero, recupera algunas prácticas en las instituciones académicas y en las comunidades estudiadas que han tratado de solventar estas problemáticas. A partir de estos tres ejes, que articulan el ensayo, analizo los siguientes procesos emblemáticos en la ética de la investigación: el riesgo/beneficio, el valor social, el consentimiento informado, la confidencialidad, la validez de los resultados y lo que denominamos como “ética desde abajo”, que incluye experiencias participativas desde las comunidades estudiadas, discutiendo sus especificidades teóricas y prácticas, así como sus alcances y limitaciones. Finalmente describo y analizo dos experiencias colegiadas en las que estuve involucrado y, a partir de lo discutido en el ensayo, propongo algunas recomendaciones dirigidas a las instituciones académicas y los científicos sociales, las comunidades con las que trabajamos y las revistas científicas. 

Los procesos de la investigación social pueden ser muy diversos, así como sus implicaciones en la ética de la investigación, lo cual comprende:

1. aspectos metodológicos que involucran la temporalidad (por ejemplo, una etnografía rápida realizada en un mes y otra durante veinte años) o al método o enfoque (fenomenológico, investigación-acción-participativa, etc.);
2. la diversidad de escenarios en los que se investiga (intervenciones humanitarias, conflictos armados, emergencias de salud, etc.);
3. el formato en el que se publican los resultados (un libro, un documental, etc.);
4. la multiplicidad de papeles que desempeña un antropólogo (investigador, activista, etc.)^1^;
5. la naturaleza de los sujetos de estudio (por ejemplo, aquellos que vulneran los derechos de otras personas y, con frecuencia, no quieren ser investigados)^4^^,^^5^; y
6. el tema de investigación (existen aspectos compartidos entre subdisciplinas y especificidades respecto a la definición y práctica de la ética de la investigación en contextos hospitalarios, implicaciones del trabajo de campo en la relación médico-paciente-institución, etc., al trabajar, por ejemplo, con poblaciones vulnerabilizadas con condiciones estigmatizantes como el VIH o la salud mental, características normativas y culturales).

Si bien muchas investigaciones sociales se desarrollan en las universidades, también son numerosas en el ámbito asociativo, en las empresas o en los ministerios. Siendo estas discusiones escasas en el contexto universitario, su presencia es aún menor en los otros ámbitos^1^^,^^6^^,^^7^. En este artículo me referiré principalmente al académico ya que es el que conozco mejor. 

En América Latina la antropología social ha tendido a considerar que los dilemas éticos en la investigación se resuelven a partir del conocimiento del investigador sobre los códigos de ética o de sus principios, y la confianza en su aplicación durante el trabajo de campo. Por lo general, con algunas excepciones, se tiende a prescindir de los comités de ética en investigación (CEI) y son escasas las discusiones institucionalizadas sobre ética por las siguientes razones:

1. la ética institucionalizada es percibida como innecesaria, al considerarse que trata de problemas políticos y no tanto éticos, que ya se encuentran incluidos en procesos de militancia y de transformación social^8^;
2. tiende a centrarse, desde una perspectiva profesionalizante y burocrática, en el quehacer antropológico (y específicamente en el levantamiento de datos) subordinando las implicaciones de los usos políticos y económicos de las investigaciones^9^;
3. no se considera adaptada al diálogo disciplinario y metodológico con las ciencias sociales al ser formulada para las ciencias de la salud^2^;
4. es innecesaria ya que existen reflexiones individuales y acuerdos institucionales tácitos^8^ o se asume que la tradición de la antropología ya incluye la deliberación sobre dilemas éticos en el trabajo de campo^1^;
5. se desconfía de los CEI por su potencial carácter prescriptivo o coercitivo^10^;
6. se entiende que su función principal es blindar a las instituciones y no proteger a las comunidades^4^;
7. no se conocen sus características^1^;
8. las instituciones en las que trabajamos no cuentan con un CEI por las razones mencionadas.

Los CEI en América Latina suelen estar vinculados a la investigación biomédica y epidemiológica, siendo obligatorios en este tipo de instituciones, y regulados por normativas y comisiones nacionales de bioética; sin embargo, no son comunes en las ciencias sociales. La mayoría de los artículos antropológicos que se publican en la región le dedican poco espacio a la ética de la investigación, tanto si han sido sometidos a un CEI como si no. Por lo general -y en el mejor de los casos- la dimensión ética se resume en un párrafo que señala que la investigación se ciñó a alguna guía internacional (como la Declaración de Helsinki) o que los datos fueron obtenidos mediante el consentimiento informado. Ahora bien ¿qué estamos entendiendo desde cada disciplina por ética en la investigación?, ¿existe una definición universal que puede aplicarse por igual en los diversos contextos socioculturales de las investigaciones? y, si no es el caso, ¿la adaptación a dichas realidades implica la relativización de sus principios hasta el punto de volverlos inoperables o sujetos a la discrecionalidad de la interpretación individual?, ¿hay definiciones y prácticas no impositivas, contextualmente adaptadas y colectivamente consensuadas? 

El origen de los CEI y de las normativas actuales sobre ética en el campo de la salud se remonta a los experimentos científicos llevados a cabo en el holocausto nazi, así como a la ausencia de una regulación previa. Las “normas éticas de experimentación en humanos” del código de Núremberg se publicaron en 1947 y subrayaban la necesidad de no producir daños al sujeto, de implementar el consentimiento informado y de la no coacción, pero desde una perspectiva centrada en la autonomía del investigador para tomar decisiones^11^. En 1964, la World Medical Association publica la “Declaración de Helsinki: Recomendaciones para orientar a los médicos en la investigación clínica”, con énfasis en el principio de beneficencia. Dicha declaración incluye por primera vez la necesidad de que existan CEI que evalúen si una investigación es o no éticamente aprobable^11^. Las décadas de 1960 y 1970 suponen un momento importante en las discusiones sobre ética en investigación en el contexto de los movimientos contraculturales, de derechos civiles y de las investigaciones no éticas como, por ejemplo, el uso del racismo en “el estudio Tuskegee de la sífilis no tratada en los hombres negros” (en el periodo 1932-1972) al explotar a la población afrodescendiente sin proporcionar consentimiento informado ni una cura^12^. En el campo de la salud, se crea el denominado código Belmont, en 1979, que acentúa la importancia del respeto para las personas, la beneficencia y la justicia, pero tiene como referente a la investigación biomédica-clínica y deja de lado necesidades específicas de la investigación social y de las ciencias del comportamiento^11^, además de omitir o no considerar la adaptación cultural, el daño comunitario o la retribución social a los participantes y sus posibles consecuencias^12^. Otro conjunto de pautas éticas internacionales para la investigación en salud fue propuesto por la Organización Mundial de la Salud (OMS), en 1982 y en 2002, en el documento denominado “Pautas éticas internacionales para la investigación relacionada con la salud en seres humanos”^13^. La Declaración Universal sobre Bioética y Derechos Humanos de la Unesco^14^ ha sido, a su vez, un código de ética internacionalmente reconocido. 

En la antropología social del contexto del norte y del Sur Global americano de la década de 1960, surgieron diversas respuestas ante el involucramiento de antropólogos, de forma encubierta, en los proyectos de contrainsurgencia impulsados por la Agencia Central de Inteligencia de EEUU en América Latina como el plan Camelot^15^. Dicho proceso recuerda el papel desempeñado por la antropología durante las dos guerras mundiales y las acciones bélicas promovidas por las agendas imperialistas de EEUU y Europa. Algunos ejemplos de ello fueron denunciados por Franz Boas^16^, quien condenó el papel del antropólogo como espía y fue censurado por dicho cuestionamiento por la American Anthropological Asociation (AAA)^17^. Otros casos refieren al uso de la etnografía para desmovilizar la resistencia de los gurkas en Nepal ante el imperio británico en el siglo XIX^18^ y convertirlos en miembros de la élite de su ejército^19^. O la implicación en labores de inteligencia para el ejército de EEUU en las guerras e invasiones en Corea, Irak y Afganistán^17^. El papel de esta antropología fue evidenciado y criticado por autores como Eric Wolf e influyó en el actual discurso ético institucionalizado en la antropología estadounidense^20^, concretamente en las declaraciones y normativas de la AAA que hoy conocemos. Cabe señalar que el primer código de ética en antropología fue declarado por la AAA en 1948 y el propuesto en 1998 es considerado como fundacional por dicha asociación^21^. Si bien este último no menciona la necesidad de que las investigaciones antropológicas deban ser evaluadas por un CEI, la declaración de 2004 remite a la “Ley federal para la protección de los sujetos humanos” de EEUU^22^, la cual se inspiró en el reporte Belmont y fue formulada para la investigación biomédica, y afirma que las investigaciones antropológicas deben ser sometidas a la consideración de un CEI con el objetivo de conocer si están o no exentas de riesgo para los participantes y, en su caso, evaluar el riesgo/beneficio^23^. 

La trayectoria de la AAA no es importante aquí porque deba ser un referente global, sino por el papel que tiene en diversas investigaciones y discusiones antropológicas sobre ética en América Latina. En la década de 1970, el caso del Colegio de Etnólogos y Antropólogos Sociales (CEAS) de México es significativo respecto a sus orígenes críticos de la contrainsurgencia y proselitismo que llevaba a cabo el Instituto Lingüístico de Verano en el país y por su obligación de supervisar que la práctica antropológica en México no defienda, asesore o justifique ningún sistema de explotación y de opresión^7^. Y algunos de los cuestionamientos que actualmente se le hacen a su proximidad con los códigos de ética de la AAA, respecto a su vocación y práctica apolítica y no vinculante^7^. 

En este sentido, la participación de antropólogos (y, entre otros, geógrafos) mexicanos en proyectos financiados por entidades como el *United States Department of Defense* es un fenómeno contemporáneo, como ha sucedido con el proyecto “México indígena” (2005-2008) inscrito en las estrategias de las Expediciones Bowman enfocadas a mapear zonas de interés militar para EEUU y que se llevó a cabo encubriendo el origen de los fondos y la transferencia de la información recabada a los militares^7^. Diversas voces cuestionaron que en este caso ni el CEAS ni la AAA hayan intervenido^7^. Cabe recordar que una de las formas de generar discusiones y acuerdos alrededor de los códigos de ética es a través de los colegios o de las asociaciones profesionales. En algunos países de América Latina, como en Chile^10^, en Brasil^1^ o en México^24^, los colegios de antropólogos han impulsado propuestas de intervención; sin embargo, al igual que ocurre con los CEI en la región^25^, no siempre cuentan con los recursos necesarios para dar seguimiento a una demanda o aplicar las decisiones adoptadas^6^^,^^8^. Es importante señalar que, si bien en la antropología es común cuestionar su aciago pasado, en una suerte de “*mea culpa fundacional*”, en otras disciplinas, como el periodismo, no existen tantas restricciones éticas para el levantamiento de datos en campo^2^. 

Las propuestas de las asociaciones, así como las declaraciones internacionales y la literatura científica, suelen identificar los siguientes procesos como constitutivos de la ética de la investigación: a) análisis favorable riesgo/beneficio y valor social, b) consentimiento informado y confidencialidad, y c) validez de los resultados. A estos procesos le añadiremos otro que denominamos como: d) la ética de la investigación desde abajo. Veamos en qué consisten y sus alcances y limitaciones para la investigación social.

## ANÁLISIS FAVORABLE RIESGO/BENEFICIO Y VALOR SOCIAL DE LA INVESTIGACIÓN

Uno de los aspectos principales en los que coinciden las publicaciones sobre la ética de la investigación -y en especial las de tipo biomédico- es la evaluación positiva riesgo/beneficio. Nuestras investigaciones deben tener más beneficios directos o indirectos para las comunidades estudiadas y para la sociedad, que posibles riesgos. Si hay potencial riesgo, los investigadores deben proponer formas de mitigarlo. Ahora bien, ¿qué se entiende por beneficio y por riesgo? El riesgo está definido por la probabilidad de que -como resultado de la investigación- a corto o largo plazo, el sujeto o comunidad estudiada sufra algún daño, lo cual refiere a todo tipo de consecuencias adversas principalmente de tipo físico, psicológico y moral, y está pensado para intervenciones biomédicas o psicológicas. Las definiciones y criterios respecto a la ética de investigación en las ciencias de la salud pueden ser distintos a los empleados en las ciencias sociales. Un ejemplo de ello es que el Reglamento de la Ley General de Salud en Materia de Investigación para la Salud en México^26^ (artículo 17, apartado I) considera que no es necesario que las investigaciones basadas en entrevistas o encuestas, que respetan la confidencialidad, sean evaluadas por un comité de ética al estar exentas de riesgo. Sin embargo, en la medida en que trabajamos con personas, toda investigación que involucre trabajo de campo (presencial o digital) implicaría algún tipo de riesgo, especialmente si son poblaciones vulnerabilizadas. Tanto una entrevista como un cuestionario o la recopilación de datos en nuestro diario de campo pueden tener implicaciones negativas para las personas participantes o llevarse a cabo sin su consentimiento informado. Se suele considerar que el trabajo retrospectivo o de archivo está exento de riesgo, sin embargo, la publicación del análisis del material obtenido en campo, años atrás, también puede tener implicaciones respecto al daño/beneficio a los protagonistas (por ejemplo, exposición a violencias, daño en su reputación, etc.). De igual forma, la experiencia del confinamiento para mitigar el riesgo de exposición y transmisión del covid-19 nos ha enseñado que el trabajo de archivo -que en un contexto análogo requiera contacto con otras personas, por ejemplo, en una biblioteca- puede implicar riesgo y debe ser evaluado. Otro aspecto para destacar es que la definición de riesgo dominante omite con frecuencia la dimensión colectiva del daño a las comunidades al centrarse en el daño y en el derecho individual. En este sentido, es frecuente que la evaluación riesgo/beneficio excluya la evaluación del posicionamiento ideológico del investigador que pueda producir daño a las personas y comunidades, así como asuma que el racismo, el neoliberalismo o el sexismo continuarán y no es necesario realizar investigaciones ni proporcionar recomendaciones para combatirlos^27^.

Cuando se trata de evaluar el potencial daño a futuro tanto del proceso como del producto de nuestras investigaciones, la tarea no es sencilla. Nadie puede conocer a ciencia cierta todos los usos e implicaciones positivas, negativas o ambivalentes de los resultados de una investigación publicada, pero sí podemos adelantar algunos de sus efectos, a partir de experiencias análogas, y tratar de mitigar riesgos. De igual forma, podemos intervenir en el debate público si percibimos una manipulación indebida de nuestras publicaciones, motivada por intereses que atenten contra los derechos de los sujetos^6^ y comunidades estudiadas. También podemos involucrarnos para que los sujetos y colectivos defiendan sus derechos ante los CEI u otras instancias cuando hayan sido perjudicados por las consecuencias de una investigación o resarcir los daños, directos o indirectos, causados por la nuestra.

El beneficio y el valor social de las investigaciones suelen referir, desde las ciencias de la salud, a que los resultados de la investigación deben, potencialmente, promover la salud futura de la comunidad y enfocarse en problemas que son relevantes para ella, evaluando una intervención terapéutica o probando una hipótesis que pueda crear un conocimiento generalizable acerca de sus resultados^28^. Si la investigación carece de valor, esta no es ética, porque expone a los sujetos innecesariamente a eventuales riesgos sin una compensación social y desperdicia tiempo y recursos^28^. Algunas de las discusiones refieren a si los beneficios se comparten entre investigadores y participantes en forma de ganancias materiales o simbólicas. Es decir, si los investigadores, la universidad o la empresa van a obtener beneficios económicos o reconocimientos académicos ¿por qué no compartirlos con las personas participantes? ¿Es suficiente con manifestar que, en función de la disponibilidad razonable, la investigación podrá potencialmente mejorar las condiciones de salud de un grupo social si no logran acceder a los medicamentos, que las personas podrán ser copropietarios de las patentes, que los resultados se publicarán en revistas de acceso abierto para que puedan ser consultados de forma libre y gratuita o que la investigación contribuirá a la creación de programas de salud pública? ¿La coautoría debe ser considerada un beneficio al incluir a nuestros participantes en los artículos? Cabe señalar que el criterio de disponibilidad razonable ha sido cuestionado en los países del Sur Global por, entre otras cosas, centrarse en el acceso (condicionado) al producto de la investigación (como una vacuna), para “evitar la explotación de la población participante” y no en otros aspectos relacionados con el beneficio comunitario como la creación de programas de salud pública, el entrenamiento de personal o la construcción de hospitales, más allá del ensayo clínico^29^. Algunas propuestas como el “Marco de beneficios justos” incluyen estas y otras recomendaciones como “compartir los beneficios económicos de la propiedad intelectual”, “fomentar el empleo y la actividad económica local”, “crear repositorios públicos de beneficios”, etc.^29^. 

Desde las ciencias sociales, diversas discusiones han apuntado la necesidad de no centrarse únicamente en el beneficio a partir de medicamentos, tratamientos o recursos económicos y entender que existen otros beneficios, como fomentar la reciprocidad a partir de la devolución del producto (documental, tesis, libro, etc.)^2^. De igual forma, si una entrevista puede tener implicaciones respecto al daño al participante también puede tener efectos terapéuticos positivos^2^. Hay que recordar, no obstante, que las relaciones recíprocas pueden ser un objetivo deseable pero no siempre alcanzable ni suponen necesariamente un beneficio compartido, y que la retribución, una transacción que compensa a los participantes por involucrarse en una investigación, no es lo mismo que el beneficio. ¿Quién nos dice que los participantes entienden nuestro libro o documental y que les interesa o beneficia? ¿Cómo se mide el beneficio de una investigación cualitativa? ¿Es necesario medirlo para que tenga valor? 

Si bien el beneficio suele ser un elemento dominante en la ética de la investigación no ocurre lo mismo con el valor social, el cual tiende a ser reducido a un sinónimo del beneficio. Existen algunas diferencias entre ambos conceptos. Una de las principales, es que el beneficio refiere a una retribución específica vinculada con los resultados de la investigación y el valor social a la generación de conocimiento que contribuya al bienestar de la sociedad. En este sentido, la evaluación riesgo/beneficio debería más bien ser riesgo/valor social considerando ir más allá de los beneficios directos como compensación por los posibles daños^30^. Por lo general, el valor social de las investigaciones ha sido menoscabado debido a las siguientes razones: a) las dificultades para definirlo o cuantificarlo; b) el individualismo explicativo del modelo biomédico; c) el olvido de los determinantes sociales de la enfermedad que ocasionan que una población se torne vulnerable; d) el conflicto de interés, de tipo económico o personal, existente en el ámbito científico, que dificulta la detección del valor social; e) la privatización del conocimiento cuando la investigación está al servicio de un sistema de mercado que privilegia un enriquecimiento sin medida por sobre el interés científico y el bienestar común^30^. Cabe preguntarse entonces ¿para quién? ¿para qué? y ¿contra quién es el valor social de una investigación? Hay que recordar que algunos de los infames experimentos realizados durante el nazismo tenían un valor social; es decir, buscaban la cura de enfermedades o posibilitaron que la ciencia avanzara, a partir de una lógica instrumental genocida que sacrificaba a ciertos sectores de la población en nombre del valor social definido por el nazismo. Un ejemplo de ello es el de la inoculación del tifus (en 1941) en los campos de concentración de Buchenwald y Natzweiler con el objetivo de crear una vacuna, que tuvo como resultado un 90% de tasa de mortalidad e involucró a la multinacional farmacéutica Bayer^31^. Este es uno de los muchos experimentos que se llevaron a cabo usando a la población prisionera como cobayas humanas. 

En la actualidad la gestión del covid-19 respecto a la desigualdad en la distribución de las vacunas, que tuvo una muy baja cobertura en muchos de los países del Sur Global lo cual comprende aquellos en cuyas poblaciones se habían ensayado, como fue el caso de Sudáfrica^32^, es un ejemplo evidente de las ganancias multimillonarias de la Big Pharma, las vidas prescindibles y la instrumentalización de la ética que aprueba dichos experimentos, pero no garantiza la redistribución justa de sus beneficios. 

Las cinco dimensiones mencionadas anteriormente, que menoscaban el valor social de las investigaciones, recuerdan que ese valor social no se entiende si la ética no es a la vez política y analiza críticamente las relaciones de poder que la definen y se ponen en juego en entramados económicos, políticos y culturales que asignan valores, distribuyen bienes y disputan sentidos. Si lo que deseamos es que el valor social se relacione con la justicia social, sería importante someter al criterio de la ética de la investigación a las agencias financiadoras de proyectos, a las instituciones académicas que reciben los fondos, a los conflictos de intereses de los investigadores (que con frecuencia son funcionales a la lógica corporativa de sus universidades), a los procesos y resultados de las investigaciones y a las editoriales y revistas científicas que operan orientaciones ideológicas y económicas específicas. Algunos de estos procesos son evaluados actualmente por CEI o comités *ad hoc*, y se han creado códigos como el *Missenden Code of Practice for Ethics and Accountability* (Código de Ética y Responsabilidad de Missenden) para enfrentar los desafíos de los fondos privados (comerciales) para la investigación en las universidades^33^. Sin embargo, no siempre incluyen otros escenarios en los que se lleva a cabo investigación (como la sociedad civil o la empresa privada) y parecen no ser todavía suficientes cuando el conflicto de interés (no declarado) es frecuente en la investigación y el diseño de políticas públicas financiadas por el sector privado^34^^,^^35^ o cuando las editoriales y revistas científicas sirven a los intereses de las grandes corporaciones de la biomedicina, al colonialismo o a la mercantilización del conocimiento^33^^,^^36^. Es respecto al valor social que la ética de la investigación, además de ayudar a reflexionar sobre el poder y sus consecuencias, requiere ser una práctica colectiva que invite a la transformación social posicionándose a partir del ¿para qué?, ¿para quién? y ¿contra quién es definida y utilizada? 

## EL CONSENTIMIENTO INFORMADO Y LA CONFIDENCIALIDAD

A diferencia del médico, del sacerdote o del abogado que reciben la petición de ayuda del cliente y tienen derecho a obtener información confidencial, el científico social solicita ayuda a su informante y le promete a cambio su responsabilidad para mantener la confidencialidad^15^. La confidencialidad es un principio, y una práctica^37^, que refiere a la garantía de que la información proporcionada por un participante en una investigación será protegida y no se revelará a terceros sin su autorización. Para ello, la obligación del investigador es implementar medidas adecuadas que preserven el derecho a su privacidad. La confidencialidad remite a las acciones del investigador y la privacidad, al derecho del sujeto a estar libre de perturbaciones o intrusiones en su vida privada^38^. Algunas de estas acciones consisten en el almacenamiento de los datos en un lugar debidamente protegido y en su anonimización. Sin embargo, los criterios para ello difieren entre las recomendaciones de la ética biomédica y las normativas propuestas por algunos colegios de antropólogos. Este es el caso de la preservación o no de las entrevistas. Mientras los comités biomédicos de ética apuntan a la destrucción de este tipo de archivos, trascurrido un tiempo determinado, algunos códigos antropológicos refieren a la obligación del investigador de preservar la información para futuros estudios^24^. A diferencia de los métodos cuantitativos, que utilizan muestras de población de gran tamaño, en las investigaciones cualitativas la identificación es mucho más sencilla. Las implicaciones negativas de una identificación no deseada pueden ser de distinta naturaleza y conllevan no solo a reflexionar sobre si incluir información que pueda identificar a nuestros informantes (en el caso de que no quieran ser identificados), también los lugares de estudio e incluso si es viable o no una investigación sin revelar el grupo, institución o contexto estudiado, lo cual involucra a su vez al tipo de producto (como un libro o un video^2^. 

Ahora bien, ¿y si de lo que se trata es precisamente de producir evidencia para demostrar la relación de las prácticas de un sujeto, grupo social o institución con la vulneración de los derechos humanos de otros?, ¿debemos entonces defender el derecho a la privacidad y apegarnos al principio de la confidencialidad? En la investigación social se asume que incluso cuando se estudia la organización social de la vulneración y a sus protagonistas, debemos preservar el anonimato de las fuentes, y los sujetos a los que aluden, y evidenciar las relaciones sociales y contextos que fomentan y reproducen dicha vulnerabilidad^4^. Incluso en los casos vinculados a la ilegalidad, la transposición entre la investigación policial y la etnográfica, al transgredir el principio de la confidencialidad, no es por lo general bienvenida^6^. En otras palabras, si lo que pedimos es información a cambio de la confidencialidad, es la credibilidad social en nuestra capacidad de asumir este principio lo que nos permite seguir trabajando. Los casos en los que se entiende que no debemos preservar la confidencialidad tienen que ver con los acuerdos previos que se hacen con la persona participante, respecto al tipo de información que proporciona, así como con una orden judicial vinculada con una legislación vigente. Si bien no es frecuente que legalmente se le exija al antropólogo revelar las fuentes, esto podría ocurrir^15^ y, a diferencia de la medicina o la abogacía, no contamos con regulaciones profesionales tan evidentes. Son diversas las investigaciones que muestran los dilemas éticos que surgen en este tipo de situaciones, cuando, por ejemplo, somos testigos de actos de violencia o de confesiones de futuros daños^39^. Algunas nos recuerdan la importancia tanto de la ética situacional^18^, y las estrategias reactivas que surgen en campo^40^, como de la necesidad de llevar a cabo acciones informadas, no siempre sencillas de dilucidar individualmente, que traten de valorar las posibles consecuencias de nuestras prácticas, minimizando daños para los sujetos y comunidades involucradas que nos comparten su intimidad, así como para nosotros mismos y para nuestra profesión. Veremos a continuación cómo esto se complejiza con las definiciones y los usos dominantes del consentimiento informado. 

El consentimiento informado busca que las personas con las que llevamos a cabo nuestras investigaciones conozcan los objetivos, métodos, riesgos (esperados o no, pero posibles), beneficios (o su ausencia), valor social, tipo de participación esperada y posibles usos de la investigación, así como los principios de voluntariedad y derecho a la información y a la confidencialidad, para poder decidir o no participar en ellas. Según las tradiciones y enfoques éticos y disciplinarios, el consentimiento y asentimiento (si se trata de participantes que legalmente no pueden proporcionar el primero) puede ser oral, registrado en audio, o escrito en una carta de consentimiento o asentimiento informado. Con algunas excepciones que lo justifiquen^41^, se recomienda su obtención al comienzo de la investigación. Si bien es un principio fundamental al que cualquier investigación debe aspirar, resulta más útil entenderlo como un proceso que como un hecho acabado. En este sentido, toda investigación tiene un componente encubierto o de consentimiento informado inacabado^4^, e incluso pueden existir investigaciones que no cumplan con el consentimiento, y esto sea lo deseable, como aquellas con personas y colectivos que, en contextos de impunidad, vulneran los derechos humanos^4^. 

Un ejemplo de la compleja articulación entre investigación etnográfica e investigación policial, y entre la adscripción académica y el activismo, refiere a la investigación encubierta de Nancy Scheper-Huges, en EEUU y Sudáfrica, sobre el tráfico de órganos^6^. Aunque esto sea un hecho que tiene que ver con las características del proceso investigativo, así como con las múltiples adscripciones referidas, lo que no es tan común es discutir qué elementos encubiertos tienen nuestras investigaciones, y si es una opción investigativa o una parte inevitable del proceso, así como para qué y respecto a qué temas y poblaciones esto se debe visibilizar o no^4^. Tres son los procesos en los que el consentimiento informado puede estar comprometido: a) durante el acceso al campo y el desarrollo del levantamiento de datos; b) en la gestión de los resultados; y c) en la temporalidad de la intención investigativa^4^. El primer caso ocurre con la negociación de unos objetivos y estrategias que cambian a lo largo del trabajo de campo o se negocian, develando parcialmente cuáles son, para asegurar la entrada al campo o únicamente se proporcionan a los porteros y no a todos los actores involucrados. Lo mismo sucede con la devolución del producto final que no se realiza en un lenguaje o formato accesible, se dirige solo a ciertos sectores de los colectivos estudiados o contiene información parcial. La temporalidad juega un papel importante cuando, por ejemplo, se llevan a cabo autoetnografías que recuperan información personal, que involucra a terceros, desprovista de intención investigativa y, por tanto, de consentimiento informado, y se convierte en “material publicable”^4^. También puede ocurrir con los nuevos usos de un material etnográfico recabado en el pasado. Algunas estrategias para afrontar esto consisten en considerar que no es suficiente con obtener el consentimiento informado una sola vez y su negociación debe ser sucesiva, lo cual incluye la devolución del producto final^2^. 

Una problemática señalada desde las ciencias sociales es que el consentimiento informado se suele entender desde la concepción de un sujeto autónomo y racional que libremente manifiesta su voluntad para participar en la investigación, sin considerar los condicionantes que pueden mediar en ello^41^. Teniendo en cuenta que nuestras investigaciones involucran con frecuencia a poblaciones vulnerables con las cuales existen diferencias de poder, el principio de voluntariedad puede ejercerse en contextos de escasa autonomía. Este es el caso, por ejemplo, de los usuarios de un servicio de salud derivados por el personal que temen sufrir repercusiones negativas. El propio formato del consentimiento puede ser percibido como un refuerzo de la desigualdad en las relaciones de poder^42^. Algunas estrategias para minimizar la mediación de las relaciones de poder en la obtención del consentimiento informado sugieren que sea obtenido a través de personas significativas para el participante y distintas al investigador^2^, o enviarlo con la suficiente antelación para que pueda ser analizado. Otras problemáticas residen en asumir las categorías “consentimiento” e “información” como universales sin una adecuación social y cultural^42^, o el lenguaje burocrático y poco comprensible de los formatos de consentimiento informado^42^, o la obligatoriedad de la firma del documento que puede poner en riesgo a nuestros informantes cuando, por ejemplo, están involucrados en actividades ilícitas^6^. Tampoco es frecuente que se expliciten todos los usos de la información obtenida ni siempre se asegura que los participantes reciban una copia de dicho consentimiento informado^27^. La forma actual de proceder parece estar más preocupada por blindar a las instituciones de posibles demandas que por el valor ético del consentimiento informado o por la apuesta por relaciones de confianza, transparencia y reciprocidad entre investigador y participantes^42^. Sin duda, todavía es un proceso que, a pesar de ser el más debatido en las ciencias sociales, requiere un mayor descentramiento del ámbito biomédico y de las ciencias del comportamiento cuantificable y una discusión más visible y colectiva desde las ciencias sociales y los métodos cualitativos. Algo similar sucede con la validez de los resultados, por lo general, concebida desde una dimensión cuantitativa, positivista y técnica que puede ocultar el componente ideológico en su evaluación y excluir o no entender las características de la investigación cualitativa. Lo discutiremos a continuación.

## VALIDEZ DE LOS RESULTADOS

La validez de los resultados de una investigación se relaciona con la ética en la medida en que puede afectar su valor social, la protección de los participantes, la credibilidad científica o el uso responsable de los recursos. Si bien la validez es un criterio compartido entre la investigación cuantitativa y la cualitativa, que refiere a si la investigación es capaz de generar evidencia fiable y precisa de aquello que pretende estudiar, existen algunas diferencias notables en cuanto a su definición y usos. Una de las principales, consiste en que la investigación cuantitativa busca la objetividad y la generalización estadística de sus hallazgos, mientras que la cualitativa, y el método etnográfico concretamente, por un lado, reconoce que los datos científicos son construcciones sociales en las cuales está involucrada la subjetividad del investigador; y, por otro lado, puede aspirar a una generalización tipológica o analítica de sus hallazgos^43^. En este sentido, la investigación cuantitativa tiende a minimizar o a controlar los sesgos, mediante la estadística (estandarización, muestreo probabilístico, grupos de control, etc.), mientras que la cualitativa asume que su tarea principal es tratar de vigilarlos, describirlos y explicarlos. Estas diferencias tienen implicaciones en la medida en la que un CEI, cuyos miembros no cuentan con experiencia en investigación cualitativa, pueden no entender, por ejemplo, el valor de la observación participante para la triangulación de los datos obtenidos en las entrevistas, o considerar que un estudio de caso, con una muestra de 20 personas, es numéricamente insuficiente para la validez de los resultados, o determinar que la técnica de “bola de nieve” no garantiza el derecho a la participación de todos los sujetos en la medida en la que direcciona la selección de los participantes^44^. Otra particularidad de la validez reside en el valor que se le atribuye a la evidencia en distintos tipos de ejercicios profesionales, como es el caso de la interlocución entre el peritaje antropológico y el derecho, cuando los antropólogos deben regirse por unos principios éticos y científicos, basados en la evidencia empírica, distintos a los abogados, que están obligados a defender a su cliente^6^.

Por último, otra dimensión problemática del uso de los criterios de validez ha sido señalada cuando sirven para ocultar las ideologías que subyacen a la aprobación o no de protocolos socialmente sensibles^44^. Por ejemplo, cuando se invoca a la objetividad científica para no validar proyectos de investigación que tienen propósitos explícitamente políticos como la lucha contra el Apartheid o la homofobia, a partir de la indagación en la discriminación institucional^44^. En este sentido, los CEI no operan en un vacío ideológico y no es ético esconderse tras la validez científica para no discutir abiertamente las ideologías^44^, prejuicios y supuestos implícitos de sus integrantes. A ello se le añade que no es tan frecuente que su implementación y funcionamiento sean investigados en profundidad^45^ ni en contextos del Sur Global como América Latina^23^. Cabe preguntarse ¿la validez de los resultados tiene que ver con el método y el diseño de la investigación o con la capacidad de ayudar a resolver los problemas a las comunidades con las que trabajamos?^46^ ¿Cómo explicamos esa validez y para quiénes son válidos o no nuestros resultados? Veremos, en el apartado siguiente, algunos de los alcances y desafíos de plantear la ética de la investigación desde las comunidades, por lo general subalternizadas, que participan en nuestras investigaciones.

## LA ÉTICA DE LA INVESTIGACIÓN DESDE ABAJO

La ética de la investigación no es definida y evaluada únicamente por los investigadores, también lo hacen las personas y colectivos que participan en nuestras investigaciones para protegerse de nosotros. Algunas comunidades, como las zapatistas en Chiapas, han considerado que los riesgos potenciales son mayores que los beneficios -por ejemplo, en el uso de la información publicada por parte de actores contrainsurgentes del Estado y/o del crimen organizado- lo que ha llevado a restringir las investigaciones en sus territorios^4^. Sin embargo, las deliberaciones que las comunidades llevan a cabo para permitirnos la entrada al campo no siempre están vinculadas a la ética de la investigación y, ante la imposición de criterios externos, en diversos contextos del Sur Global, algunas comunidades han considerado importante incorporar respuestas al respecto. Una de ellas ha consistido en crear CEI en las comunidades, que incluyen definiciones y usos de la ética producidas por la academia y por la comunidad^27^^,^^47^ y, a diferencia de otro tipo de grupos comunitarios, revisan los protocolos desde una perspectiva ética, con capacitación específica en ética y métodos de investigación^27^. Otra de las formas de coproducir “ética desde abajo” ha consistido en la participación en los CEI de representantes de las comunidades estudiadas. Son diversas las declaraciones internacionales que señalan la importancia de ello; sin embargo, no siempre ocurre ni se especifica por qué es importante su participación, cuáles serán sus funciones o si son considerados como pares en la toma de decisiones del CEI^48^. Algunas de las problemáticas detectadas son las definiciones múltiples de los conceptos de comunidad y representación, la incomprensión de la importancia de su desempeño o la desigualdad de poder respecto a los profesionales que forman parte de los CEI^3^^,^^49^. 

Otra de las modalidades existentes “desde abajo” es la ética participativa en las investigaciones basadas en la investigación acción participativa (IAP) o en enfoques que, con características diferenciadas, emanan de ella como, por ejemplo, la “*community based participatory research*” frecuente en el campo de la salud. La investigación acción participativa, por sus características metodológicas y epistemológicas, incluye reflexiones sobre ética de la investigación que involucran la participación de las comunidades. Esto ocurre en la coparticipación de los investigadores y los miembros de dichas comunidades en las demandas de la investigación, en el proceso, en los resultados, en los productos y en sus usos, lo cual comprende a las definiciones y acciones respecto al consentimiento informado, el riesgo/beneficio/valor social o la validez de los datos. Algunas orientaciones de la investigación acción participativa enfatizan en la necesidad de la justicia epistémica a partir del reconocimiento y la valoración de epistemologías, conocimientos, teorías y experiencias de las personas participantes y su reivindicación ante la hegemonía tanto de la ética y las teorías del Norte Global^49^ como de la academia del Sur Global que, a su vez, reproduce desigualdades. No obstante, diversas publicaciones han señalado que son necesarias guías específicas y marcos conceptuales para este tipo de investigaciones, ya que se asume que se cumplirá con la inclusión, mutualidad, beneficio y control comunitario, pero no cómo se hará y comprobará que se llevó a cabo^46^. Una pregunta que surge es que, si nos basamos en lo que definen las comunidades con las que trabajamos como ético, esto puede ser problemático, ya que lo que una comunidad ve como ético otra no lo considera ético^50^^)*.*^ También sucede con los miembros dentro de una misma comunidad cuando trabajamos, por ejemplo, con grupos sociales específicos vulnerados por la propia comunidad, que no tienen capacidad o tienen una capacidad limitada de movilización colectiva dentro de esa comunidad. Otra característica de la investigación acción participativa es que no siempre cumple con los tres procesos y con frecuencia es más investigación que acción o más acción que participación o investigación. De igual forma, la participación es definida de maneras muy diversas y en ocasiones las definiciones de los investigadores no coinciden con las de las comunidades estudiadas^51^, con implicaciones éticas en los principios preestablecidos que deben adaptarse, negociarse y explicarse. Como respuesta a los problemas que encierra la ética pensada desde afuera de las comunidades, en los últimos años se han llevado a cabo recomendaciones sobre ética y participación social (*community engagement*), como una de las expresiones de la investigación acción participativa, en contextos investigativos diversos como la ayuda humanitaria^52^, en forma de lineamientos y competencias éticas plasmadas en códigos, guías u otro tipo de formatos. Si la ética en la investigación desde abajo cuenta con diversas experiencias como las descritas ¿qué estamos haciendo en nuestras instituciones para discutir y decidir colectivamente sobre ética desde las ciencias sociales?

## Algunas experiencias institucionales en México

Desde hace varios años he participado en la implementación y desarrollo de dos experiencias institucionales de evaluación de aspectos éticos de la investigación antropológica en el Centro de Investigaciones y Estudios Superiores en Antropología Social (CIESAS), de México. La primera es un CEI en salud, que hace aproximadamente diez años propusimos Graciela Freyermuth, Paola Sesia y yo, quienes estamos adscritos a dicha institución. Su puesta en práctica surgió ante la necesidad de contar con un espacio colegiado de discusión y evaluación de la ética de la investigación, en el campo de la salud, ante las limitaciones que percibíamos en el ejercicio individual de una evaluación de esta naturaleza, y la creciente demanda de diversas instituciones y revistas científicas de una evaluación formal de nuestros protocolos para realizar trabajo de campo, participar en investigaciones interinstitucionales o publicar. Otra de las razones era no depender de los comités de ética biomédicos, por su limitada sensibilidad a la investigación social, al no contar con un CEI en nuestra institución. Para el diseño y la implementación del CEI en CIESAS, el cual coordiné desde 2020 hasta 2024, se creó un protocolo a partir de la legislación existente -concretamente la Ley General de Salud^26^ y la Combioética^53^- y se adaptaron los lineamientos a la investigación de tipo antropológico, considerando algunas de las cuestiones aquí discutidas, a la par que se llevaron a cabo los pasos para su institucionalización. En este proceso participaron colegas de CIESAS y de otras instituciones mexicanas como el Instituto Nacional de Salud Pública. Con el objetivo de que no tuviera un carácter coercitivo, se instauró para aquellos que, de forma voluntaria, estuvieran interesados en recibir consejos o una evaluación formal de sus protocolos. Las características del CEI son parecidas a otros existentes en México, con la diferencia de que hay siempre expertos en investigación social cualitativa. Dedicado exclusivamente al campo de la salud, fue recibiendo paulatinamente solicitudes de investigadores del CIESAS, o colaboradores externos, en otras áreas, lo cual ha implicado reflexionar sobre su estructura y características. A pesar de su juventud, ha evaluado minuciosamente diversos protocolos proporcionando insumos, adaptados a la naturaleza cualitativa de nuestras investigaciones, para el apego a la ética de la investigación social en salud, y recopilando a su vez materiales para la actualización de sus miembros. Este no es el único CEI en el ámbito académico mexicano adaptado a las ciencias sociales, sin embargo, son mucho menos numerosos que los existentes en las instituciones destinadas a la investigación en salud. A modo de autocrítica, y entendiendo que la primera etapa concentró los esfuerzos en tratar de crear las bases para su funcionamiento y formalización, considero que sería conveniente: a) involucrar en las evaluaciones a los grupos sociales que participan en las investigaciones, b) visibilizar la importancia del trabajo del CEI con el objetivo de fomentar la participación de la comunidad académica en su desarrollo y, c) facilitar espacios de discusión sobre las evaluaciones de los protocolos, con sus autores, que no las reduzcan a un intercambio de correos y documentos. 

La segunda experiencia en la que he participado es la Comisión de Riesgo creada en 2020, y actualmente activa, como una comisión *ad-hoc* del Colegio Académico del Posgrado en Antropología del CIESAS, en Ciudad de México. Dicha comisión ha sido coordinada por Susann Vallentine Hjorn y en su implementación y desarrollo también han participado Hiroko Asakura y Carolina Robledo Silvestre, las tres investigadoras adscritas al CIESAS. Su objetivo principal es realizar recomendaciones a partir de las evaluaciones de riesgo del trabajo de campo de los estudiantes del posgrado. Con la llegada del covid-19 fui invitado a participar con el objetivo de involucrarme en la evaluación del riesgo y mitigación de la transmisión del virus. Si bien la Comisión de Riesgo no fue concebida como un CEI, muchas de sus recomendaciones se articulan con la ética de la investigación social. Su carácter consultivo, ha sido importante para orientar a partir de recomendaciones individuales y colectivas (lo cual incluye su participación en talleres de riesgo y el análisis y seguimiento de incidentes posteriores al trabajo de campo) en temáticas específicas como, por ejemplo, la exposición a violencias o la salud. En el campo de la ética, y respecto al covid-19 específicamente, la Comisión de Riesgo no solo ha recomendado formas de mitigación del riesgo para los estudiantes, los participantes en la investigación y sus familiares y contactos, también ha desalentado que se lleven a cabo investigaciones cuyo potencial daño era mayor al beneficio, sugiriendo modalidades virtuales o a distancia (en contextos de confinamiento voluntario).

Si bien estas experiencias no constituyen soluciones que resuelvan siempre y en cualquier contexto todos los dilemas de la ética de la investigación ni garanticen el comportamiento ético de las personas, sí suponen un acompañamiento colectivo que trata de fomentar la crítica constructiva, la autonomía investigativa y, a su vez, supervisar las relaciones de poder, entre los involucrados en una investigación y las comunidades a las que pertenecen, y su potencial daño. 

Propondremos, a continuación, algunas recomendaciones a partir de las experiencias y publicaciones discutidas en el presente texto para los científicos sociales y las comunidades con las que trabajan, para los CEI y las instituciones en las que operan y para las revistas científicas.

### Algunas recomendaciones para una ética de la investigación social con menos ausencias y más posibilidades

#### Para los científicos sociales y las comunidades con las que trabajan

- Promover, en las instituciones, espacios de discusión colectiva sobre la ética de la investigación, que incluyan a estudiantes, investigadores y miembros de las comunidades sujeto de estudio. Se trataría de compartir experiencias, así como material para ser revisado y discutido con el objetivo de mejorar las deliberaciones individuales o colectivas en nuestras investigaciones. A partir de ello, proponer recomendaciones concretas en formatos accesibles.
- Impulsar comités colegiados, con un carácter consultivo, para realizar evaluaciones formales de los protocolos de investigación. Estos comités deben estar adaptados a las características de la investigación cualitativa y cuantitativa en ciencias sociales, con criterios que permitan discutir con las guías existentes, así como con un funcionamiento transparente, rotatorio y que involucre la participación voluntaria, como evaluadores o evaluados, de la comunidad académica.
- Considerar a las personas participantes en las investigaciones como sujetos con derechos individuales y comunidades con derechos colectivos que tienen saberes que pueden contribuir a la práctica ética y a las recomendaciones de los CEI.
- Realizar investigaciones cualitativas sobre el funcionamiento de los CEI en instituciones de salud, universidades, empresas, organismos internacionales o ministerios públicos para conocer los alcances y las limitaciones de dichos recursos e invitar a la discusión.
- Sería conveniente que los equipos de investigación privilegien publicar sus resultados en revistas que promuevan el acceso libre y gratuito a los contenidos publicados, y habiliten el acceso a la población general y especialmente a aquellas comunidades que presentan mayores desventajas socioeconómicas y digitales para acceder a la información científica.
- Recomiendo que, a pesar de lo señalado anteriormente, los miembros de las comunidades estudiadas creen sus propios CEI para evaluar nuestras investigaciones y protegerse de sus potenciales riesgos, generando sus lineamientos con competencias éticas (y recursos legales para defenderlas) en guías o reglamentos que puedan compartir y discutir con otras comunidades.

#### Para los CEI y las instituciones en las que operan

- Considerar que la ética en investigación no se reduce a la operación del CEI para aprobar investigaciones, ni a la obtención del consentimiento informado para recolectar datos, sino a un proceso de reflexión y discusión continua que atraviesa a toda la investigación, desde su concepción hasta los productos.
- Incluir a expertos en métodos cualitativos de investigación social.
- Promover la participación de los representantes de las comunidades estudiadas en los CEI, comisiones u otro tipo de espacios colegiados.
- Cuando no sea evidente en el protocolo de investigación evaluado, plantear preguntas a los investigadores que incluyan para qué y para quién y no solo el cómo, qué, cuándo o quién.
- Impulsar principios, capacitaciones y guías claras respecto a aspectos sensibles relacionados con poblaciones vulnerables y derechos humanos, así como a los diversos tipos de métodos de investigación y tradiciones epistemológicas existentes.
- Incluir a especialistas con experiencia en ética comunitaria, en investigación acción participativa y en participación social y, de ser posible, en los contextos en los que se realizarán las investigaciones
- Fomentar espacios de discusión constructiva sobre ética con los equipos de investigación con el objetivo de que sus evaluaciones no sean un mero proceso burocrático ni un ejercicio autoritario.
- Garantizar que los CEI tengan recursos para realizar su labor de forma continua, y puedan dar seguimiento a las investigaciones cuyos protocolos fueron evaluados.
- Examinar si los protocolos de investigación se inscriben en valores e ideologías (mediadas por los investigadores, sus instituciones y/o las agencias financiadoras), que puedan dañar a los colectivos participantes y proponer estrategias para su afrontamiento.
- Reflexionar colectivamente sobre la influencia de las ideologías, valores y supuestos implícitos de los miembros de los CEI (y sus comunidades e instituciones de adscripción) en la definición de lo ético y en el papel que desempeñan dichos procesos y sus contradicciones en el funcionamiento del CEI.
- Implementar mecanismos eficaces que eviten que los CEI se conviertan en un espacio de poder en el que se enmascaran los conflictos de interés o las agendas propias mientras se evidencian las ajenas.
- Proponer discusiones integradas a los CEI, o a otros comités específicos, que analicen la ética de las agencias e instituciones financiadoras de investigación respecto a sus agendas y potenciales usos de los productos; lo cual incluye a los posibles conflictos de interés de los investigadores y de las agencias financiadoras.

#### Para las revistas científicas

- Sugerir que los artículos presenten el aval de un CEI o una instancia análoga y, si su institución no cuenta con uno o los autores decidieron no someterlo, información sobre las razones por las que no fue evaluado y respecto a las acciones llevadas a cabo para que la investigación cumpla con los criterios éticos necesarios. Proporcionar un espacio adicional, a la extensión máxima autorizada, para que el autor pueda describirlo.
- Si es necesario, solicitar material complementario para comprobar que la investigación se apegó a los criterios éticos requeridos. Por ejemplo, los formatos de consentimiento informado o información adicional respecto al resguardo de los datos.
- Incorporar en los consejos editoriales de las revistas a expertos en ética de investigación social que puedan asesorar al cuerpo editorial.
- Promover espacios editoriales de discusión sobre la ética de la investigación, sus principios universales, sus especificidades locales, los campos de poder que determinan qué es ético o no, las implicaciones y características en cada disciplina, las prácticas normativas o alternativas que pueden resultar eficaces, etc.
- Revisar críticamente si sus publicaciones han promovido el clasismo, el racismo, el sexismo, la xenofobia, la homofobia, el capacitismo o cualquier otro tipo de opresión, así como prácticas extractivas o de despojo de ecosistemas, regiones o conocimientos y saberes. En caso afirmativo, llevar a cabo medidas de reparación promoviendo espacios y circuitos de publicación que permitan visibilizar el daño, a partir de las voces de los afectados, y las formas de afrontarlo.

## CONSIDERACIONES FINALES

En este artículo se han discutido algunas de las ausencias, urgencias y posibilidades de la ética en la investigación social en América Latina, con diversos ejemplos respecto a la antropología y la salud. Para ello se han propuesto tres ejes de análisis que refieren a las escasas discusiones colectivas e institucionalizadas y a sus repercusiones negativas en nuestro desempeño profesional; a la exclusión de las especificidades de la investigación social y del análisis de las relaciones de poder desde las imposiciones de las ciencias de la salud del Norte Global; y a la existencia de algunas prácticas en las instituciones académicas y en las comunidades estudiadas que han tratado de solventar estas problemáticas. En este sentido, la ética en la investigación es siempre política en la medida en que debería reflexionar sobre las relaciones desiguales de poder y sus consecuencias respecto a la definición de lo ético, la elección de aquello que se estudia, cómo y para qué se estudia, actuando colectivamente sobre las posibilidades para su transformación como la base del valor social, la evaluación del riesgo/beneficio, el consentimiento informado, la confidencialidad y la validez de los resultados. Para ello es fundamental cultivar el territorio diverso de la ética del cuidado^54^ ante el monocultivo de la ética de la productividad^55^.
