# Supplementary material for: Ethics in social research, its absences, urgencies, and possibilities: Critical proposals from Latin America
Source: Salud Colect. 2025 Aug 8;21:e5759. [Article in Spanish] doi: 10.18294/sc.2025.5759 (PMC12718610; doi:10.18294/sc.2025.5759)
Supplement: Translation

# Translation: Ethics in social research, its absences, urgencies, and possibilities: Critical proposals from Latin America

**Authors:** Rubén Muñoz Martínez

**Affiliations:** 1 Doctor of Social Anthropology. Senior researcher, Centro de Investigaciones y Estudios Superiores en Antropología Social (CIESAS), Mexico City, Mexico. rubmuma@hotmail.com

**Keywords:** Research Ethics, Research Ethics Committees, Social Sciences

## Abstract

Ethics in qualitative social research has particular features that distinguish it from the model formulated in the Global North for the health sciences, which is characterized by a universalist, quantitative, clinical approach and a limited critical perspective on its role in power relations. In Latin American social anthropology-similarly to other social sciences-there are few institutionalized collective discussions about how ethics is being understood, excluded, and practiced in research processes. In this essay, I examine these issues in the Latin American context, along with some academic and community-based responses. I analyze key dimensions such as risk/benefit, social value, informed consent, confidentiality, validity of results, and the ethics proposed by communities themselves to protect against researchers. I also draw on two institutional experiences in which I participated, raising questions and possibilities through examples from the fields of anthropology and health.

## INTRODUCTION

The aim of this article is to raise a series of questions and discussions regarding research ethics in qualitative social research, drawing on examples from disciplinary and thematic fields such as anthropology and health. This piece emerges from what I identify as absences, demands, and responses across various professional circuits in the social sciences in Latin America, both in how ethics is defined and how it is approached. The analysis is structured around three core axes.

First, the article addresses the limited institutionalized collective discussion regarding ethics in qualitative social research and its negative repercussions on our professional practice. This is especially relevant when considering the limitations of relying solely on individual deliberation or on tacit agreements within the communities from which research is conducted.

Second, it examines the potential imposition of hegemonic regulatory frameworks derived from the biomedical field (in the Global North). These frameworks, grounded in a universalist perspective^1^^,^^2^, proceduralism, and principlism^3^, and predominantly quantitative-experimental-clinical in orientation, often fail to consider -or do so in a restricted way- the specificities of other types of research and the political dimension of ethics, particularly in relation to its role in power dynamics.

Third, the article highlights some of the practices developed within academic institutions and research communities aimed at addressing these issues. Building on these three axes, I analyze the following key processes in research ethics: risk/benefit, social value, informed consent, confidentiality, validity of results, and what I refer to as “ethics from below.” This latter concept encompasses participatory experiences emerging from within the communities being studied, discussing their theoretical and practical specificities, as well as their scope and limitations.

Finally, I describe and analyze two collegial experiences in which I was directly involved. Based on the discussions presented throughout the essay, I offer a series of recommendations directed at academic institutions and social scientists, the communities with whom we work, and scholarly journals.

Social research processes can vary widely, as can their implications for research ethics. These include:

- methodological aspects involving temporality (for example, a rapid ethnography conducted over one month versus one carried out over twenty years), or the method or approach used (phenomenological, participatory action research, etc.);
- the diversity of research settings (humanitarian interventions, armed conflicts, health emergencies, etc.);
- the format in which results are disseminated (a book, a documentary, etc.);
- and the multiple roles an anthropologist may play (researcher, activist, etc.);^1^
- the nature of the research subjects (for example, those who infringe on the rights of others and who often do not wish to be studied);^4^^,^^5^
- the research topic itself (there are shared elements across sub-disciplines, but also specific issues regarding how research ethics is defined and practiced in hospital settings, the implications of fieldwork in the doctor-patient-institution relationship, etc., particularly when working with marginalized populations affected by stigmatizing conditions such as HIV or mental health issues, which carry both normative and cultural implications).

Although much social research is carried out in universities, a significant portion also takes place in civil society organizations, private companies, or government ministries. Given that discussions on research ethics are already scarce within academic institutions, they are even less common in these other settings.^1^^,^^6^^,^^7^ In this article, I focus primarily on the academic sphere, as it is the one I am most familiar with.

In Latin America, social anthropology has tended to assume that ethical dilemmas in research are resolved through the researcher’s knowledge of ethical codes or principles and their application during fieldwork. Generally -though there are some exceptions- Research Ethics Committees (RECs) are not involved, and institutionalized discussions on research ethics remain limited, for the following reasons:

- Institutionalized ethics is often perceived as unnecessary, based on the belief that it addresses political rather than ethical issues, which are already embedded in activist processes and social transformation initiatives;^8^
- From a professionalized and bureaucratic perspective, ethical oversight tends to focus narrowly on anthropological practice (specifically data collection), while subordinating concerns regarding the political and economic uses of research;^9^
- Ethical review protocols are not seen as suited to disciplinary and methodological dialogue with the social sciences, given that they were originally designed for the health sciences;^2^
- They are considered unnecessary because individual reflections and tacit institutional agreements^8^ are assumed to suffice, or because the anthropological tradition is believed to already incorporate deliberation on ethical dilemmas encountered during fieldwork;^1^
- RECs are mistrusted due to their potentially prescriptive or coercive nature;^10^
- They are often seen as mechanisms that serve primarily to shield institutions rather than to protect communities;^4^
- Their characteristics are often unknown;^1^
- Many institutions where we work do not have RECs, for the reasons mentioned above.

RECs (Research Ethics Committees) in Latin America are usually linked to biomedical and epidemiological research, being mandatory in this type of institution and regulated by national bioethics regulations and commissions. However, they are not common in the social sciences. Most anthropological articles published in the region devote little space to research ethics, whether or not they have been reviewed by a REC. Generally -and in the best-case scenario- the ethical dimension is summarized in a paragraph stating that the research adhered to some international guideline (such as the Declaration of Helsinki) or that the data were obtained through informed consent.

Now then, what are we understanding by research ethics from each discipline? Is there a universal definition that can be equally applied in the diverse sociocultural contexts of research? And if that is not the case, does adaptation to these realities imply a relativization of its principles to the point of rendering them inoperable or subject to the discretion of individual interpretation? Are there non-impositional definitions and practices, contextually adapted and collectively agreed upon?

The origin of Research Ethics Committees (RECs) and current regulations on ethics in the field of health dates back to the scientific experiments carried out during the Nazi Holocaust, as well as to the absence of prior regulation. The “ethical standards for experimentation on humans” of the Nuremberg Code were published in 1947 and emphasized the need to avoid harm to the subject, to implement informed consent, and to prevent coercion, but from a perspective centered on the autonomy of the researcher in decision-making.^11^

In 1964, the World Medical Association published the “Declaration of Helsinki: Recommendations to Guide Physicians in Clinical Research,” with an emphasis on the principle of beneficence. This declaration included, for the first time, the requirement that RECs evaluate whether or not a research project is ethically approvable.^11^

The 1960s and 1970s marked an important moment in discussions on research ethics, in the context of countercultural movements, civil rights, and unethical research studies, such as the use of racism in “The Tuskegee Study of Untreated Syphilis in Black Men” (conducted between 1932 and 1972), which exploited the African-descendant population without providing informed consent or a cure.^12^

In the health field, the so-called Belmont Report was created in 1979. It emphasized the importance of respect for persons, beneficence, and justice. However, it is based on biomedical-clinical research and neglects the specific needs of social research and the behavioral sciences,^11^ as well as omitting or failing to consider cultural adaptation, community harm, or the social return to participants and its possible consequences.^12^

Another set of international ethical guidelines for health research was proposed by the World Health Organization (WHO), in 1982 and again in 2002, in the document titled *International Ethical Guidelines for Health-Related Research Involving Humans*.^13^ The *Universal Declaration on Bioethics and Human Rights* by UNESCO^14^ has also become an internationally recognized code of ethics.

In the social anthropology of the North American and Global South contexts of the 1960s, various responses emerged to the covert involvement of anthropologists in counterinsurgency projects promoted by the U.S. Central Intelligence Agency in Latin America, such as the Camelot Plan.^15^ This process recalls the role played by anthropology during the two World Wars and in the military actions promoted by the imperialist agendas of the United States and Europe. Some examples were denounced by Franz Boas,^16^ who condemned the role of the anthropologist as a spy and was censured for this criticism by the American Anthropological Association (AAA).^17^ Other cases include the use of ethnography to demobilize Gurkha resistance in Nepal against the British Empire in the 19th century^18^ and to integrate them into the elite ranks of its army,^19^ as well as involvement in intelligence work for the U.S. military during the wars and invasions in Korea, Iraq, and Afghanistan.^17^ The role of this anthropology was documented and criticized by authors such as Eric Wolf, influencing the current institutionalized ethical discourse in U.S. anthropology,^20^ particularly in the AAA’s statements and regulations as we know them today. It should be noted that the AAA’s first code of ethics was issued in 1948, and the 1998 version is considered foundational by the association.^21^ Although this latter version does not mention the need for anthropological research to be reviewed by a Research Ethics Committee (REC), the 2004 statement refers to the U.S. “Federal Policy for the Protection of Human Subjects”,^22^ which was inspired by the Belmont Report and formulated for biomedical research. It states that anthropological research should be submitted to an REC to determine whether it is exempt from risk for participants and, if not, to assess the risk/benefit ratio.^23^

The significance of the AAA’s trajectory here does not lie in its status as a global benchmark, but in its role in various anthropological research and debates on ethics in Latin America. In the 1970s, the case of the Colegio de Etnólogos y Antropólogos Sociales (CEAS) of Mexico is particularly relevant due to its critical origins in relation to counterinsurgency and the proselytizing activities carried out by the Summer Institute of Linguistics in the country, and its mandate to ensure that anthropological practice in Mexico does not defend, advise, or justify any system of exploitation or oppression.^7^ At the same time, questions have been raised about its current proximity to the AAA’s codes of ethics, especially regarding its apolitical and non-binding vocation and practice.^7^^)^

In this regard, the participation of Mexican anthropologists (and, among others, geographers) in projects funded by entities such as the United States Department of Defense is a contemporary phenomenon, as in the case of the “México Indígena” project (2005-2008), framed within the Bowman Expeditions’ strategies aimed at mapping areas of military interest for the U.S. This project was carried out while concealing the origin of the funding and the transfer of collected information to the military.^7^ Several voices criticized the fact that, in this case, neither the CEAS nor the AAA intervened.^7^ It is worth recalling that one way to foster discussions and agreements around codes of ethics is through professional associations or colleges. In some Latin American countries, such as Chile,^10^ Brazil,^1^ and Mexico,^24^ anthropologists’ professional associations have promoted proposals for intervention. However, as with RECs in the region,^25^ they do not always have the resources needed to follow up on a complaint or enforce the decisions made.^6^^,^^8^ It is important to note that, while in anthropology it is common to critically address its troubled past, something akin to a “foundational mea culpa”, in other disciplines, such as journalism, there are far fewer ethical restrictions for collecting data in the field.^2^

The proposals put forward by professional associations, as well as international statements and the scientific literature, often identify the following processes as constitutive of research ethics: a) a favorable risk/benefit ratio and social value, b) informed consent and confidentiality, and c) validity of results. To these, we add another process, which we call: d) research ethics from below. Let us examine what these entail, along with their scope and limitations for social research.

## FAVORABLE RISK/BENEFIT RATIO AND SOCIAL VALUE OF RESEARCH

One of the main aspects on which publications on research ethics -particularly in the biomedical field- tend to agree is the positive evaluation of the risk/benefit ratio. Our research should yield more direct or indirect benefits for the studied communities and for society than potential risks. If there is a potential risk, researchers should propose strategies to mitigate it. But what is meant by *benefit* and by *risk*? Risk is defined as the likelihood that, as a result of the research, the subject or community under study will suffer harm in the short or long term. This refers to all types of adverse consequences -primarily physical, psychological, and moral- and is conceptualized with biomedical or psychological interventions in mind.

Definitions and criteria for research ethics in the health sciences may differ from those used in the social sciences. For example, Mexico’s *Reglamento de la Ley General de Salud en Materia de Investigación para la Salud*^26^ (Article 17, Section I) states that studies based on interviews or surveys that respect confidentiality need not be reviewed by a Research Ethics Committee (REC) since they are considered risk-free. However, insofar as we work with people, any research involving fieldwork -whether in person or digital- implies some type of risk, particularly when dealing with vulnerable populations. Even an interview, a questionnaire, or the collection of data in a field diary can have negative implications for participants or be conducted without their informed consent.

Retrospective or archival work is often considered risk-free; however, the publication of analyses based on material gathered in the field years earlier can also entail harm/benefit considerations for the individuals involved (for instance, exposure to violence, reputational damage, etc.). Likewise, the experience of lockdowns to mitigate the risk of exposure to and transmission of COVID-19 has taught us that archival work -when conducted in an analogous context that requires contact with other people, for example, in a library- can involve risk and should be evaluated.

Another point worth emphasizing is that the dominant definition of risk frequently overlooks the collective dimension of harm to communities by focusing solely on individual harm and rights. In this regard, it is common for risk/benefit assessments to exclude consideration of the researcher’s ideological positioning that could harm individuals and communities, and to assume that racism, neoliberalism, or sexism will persist without requiring research or recommendations aimed at combating them.^27^

When it comes to assessing the potential future harm arising from both the process and the product of our research, the task is far from simple. No one can fully anticipate all the possible uses and positive, negative, or ambivalent implications of the findings of a published study. However, we can anticipate some of their potential effects -drawing on analogous experiences- and attempt to mitigate risks. Likewise, we can intervene in public debate if we detect an improper manipulation of our publications motivated by interests that undermine the rights of the individuals^6^ and communities studied. We can also become involved to support individuals and collectives in defending their rights before RECs or other bodies when they have been harmed by the consequences of a study, or to remedy direct or indirect harm caused by our own research.

In the health sciences, the benefit and social value of research generally refer to the idea that research outcomes should, potentially, promote the future health of the community and address problems relevant to it -by evaluating a therapeutic intervention or testing a hypothesis capable of generating generalizable knowledge about its outcomes.^28^ If research lacks value, it is not ethical, as it exposes participants to potential risks without social compensation and wastes time and resources.^28^

Some debates center on whether benefits should be shared between researchers and participants in the form of material or symbolic gains. That is, if researchers, the university, or the company will obtain economic profits or academic recognition, why should these not be shared with the participants? Is it enough to state that, subject to reasonable availability, research could potentially improve the health conditions of a social group if they cannot access the medication; that participants could be co-owners of patents; that results will be published in open-access journals so they can be freely consulted; or that the research will contribute to the creation of public health programs? Should co-authorship be considered a benefit when including participants in scholarly articles?

It should be noted that the “reasonable availability” criterion has been questioned in countries of the Global South, among other reasons, for focusing on (conditional) access to the product of the research -such as a vaccine- in order to “avoid the exploitation of the participating population,” while neglecting other aspects related to community benefit. These may include the creation of public health programs, training of local personnel, or construction of hospitals beyond the clinical trial itself.^29^ Some proposals, such as the “Fair Benefits Framework,” incorporate these and other recommendations, including “sharing the economic benefits of intellectual property,” “promoting local employment and economic activity,” and “creating public benefit repositories,” among others.^29^

From the perspective of the social sciences, various debates have underscored the need to avoid focusing exclusively on benefits derived from medications, treatments, or financial resources, and to recognize the existence of other forms of benefit, such as fostering reciprocity through the return of the research product (e.g., documentary, thesis, book).^2^ Likewise, if an interview can have implications in terms of potential harm to the participant, it can also have positive therapeutic effects.^2^ It should be remembered, however, that reciprocal relationships may be a desirable objective but are not always attainable, nor do they necessarily imply a shared benefit, and that remuneration -a transaction compensating participants for their involvement in a study- is not the same as benefit. Who can assure us that participants understand or are interested in our book or documentary, or that these in fact benefit them? How can the benefit of qualitative research be measured? And is it necessary to measure it in order for it to have value?

While benefit is often a dominant element in research ethics, the same cannot be said for social value, which tends to be reduced to a synonym for benefit. There are, however, some distinctions between the two concepts. One of the main differences is that benefit refers to a specific retribution linked to the research outcomes, whereas social value refers to the generation of knowledge that contributes to the well-being of society. In this sense, risk/benefit assessment should rather be reframed as risk/social value assessment, going beyond direct benefits as compensation for potential harm.^30^

In general, the social value of research has been undermined for several reasons: a) difficulties in defining or quantifying it; b) the explanatory individualism of the biomedical model; c) neglect of the social determinants of disease that render a population vulnerable; d) conflicts of interest -economic or personal- within the scientific field, which hinder the detection of social value; and e) the privatization of knowledge when research is placed at the service of a market system that privileges unlimited profit over scientific interest and the common good.^30^ This prompts us to ask: *for whom?*, *for what purpose?*, and *against whom* is the social value of research defined? It should be recalled that some of the infamous experiments conducted under Nazism claimed to have social value -that is, they sought cures for diseases or contributed to scientific advancement- but did so within a genocidal instrumental logic that sacrificed certain sectors of the population in the name of a social value defined by the Nazi regime. An example of this is the typhus inoculation (1941) in the Buchenwald and Natzweiler concentration camps, aimed at developing a vaccine. The experiment resulted in a 90% mortality rate and involved the multinational pharmaceutical company Bayer.^31^ This was one of many experiments in which prisoners were used as human guinea pigs.

In the present day, the management of COVID-19 in relation to vaccine distribution inequalities -marked by extremely low coverage in many countries of the Global South, including some whose populations had been involved in vaccine trials, such as South Africa-^32^^)^ offers a clear example of the multibillion-dollar profits of Big Pharma, the existence of expendable lives, and the instrumentalization of ethics that endorses such experiments but fails to ensure the fair redistribution of their benefits.

The five dimensions mentioned above, which undermine the social value of research, serve as a reminder that such value cannot be understood unless ethics is, at the same time, political and critically analyzes the power relations that define it and come into play within economic, political, and cultural frameworks that assign value, distribute resources, and dispute meanings. If the aim is to link social value with social justice, it would be important to subject to the scrutiny of research ethics the funding agencies for research projects, the academic institutions that receive such funds, the conflicts of interest of researchers (which are often aligned with the corporate logic of their universities), the processes and results of research, as well as the publishers and scientific journals that operate under specific ideological and economic orientations.

Some of these processes are currently evaluated by Research Ethics Committees (RECs) or *ad hoc* committees, and codes such as the Missenden Code of Practice for Ethics and Accountability have been developed to address the challenges posed by private (commercial) research funding in universities.^33^ However, they do not always encompass other settings in which research is conducted (such as civil society or the private sector) and still seem insufficient when undeclared conflicts of interest are common in research and in the design of public policies funded by the private sector,^34^^,^^35^ or when scientific publishers and journals serve the interests of large biomedical corporations, colonialism, or the commodification of knowledge.^33^^,^^36^. With regard to social value, research ethics, in addition to fostering reflection on power and its consequences, must become a collective practice that invites social transformation, positioning itself in relation to the questions: for what purpose, for whom, and against whom is it defined and used?

## INFORMED CONSENT AND CONFIDENTIALITY

Unlike a physician, priest, or lawyer -who receives a request for help from a client and has the right to obtain confidential information- the social scientist requests assistance from an informant and, in return, promises to assume responsibility for maintaining confidentiality.^15^ Confidentiality is both a principle and a practice,^37^ referring to the guarantee that the information provided by a research participant will be protected and will not be disclosed to third parties without their authorization. To this end, it is the researcher’s obligation to implement adequate measures to safeguard the participant’s right to privacy. Confidentiality concerns the researcher’s actions, while privacy refers to the participant’s right to be free from disturbances or intrusions into their private life.^38^ Such actions may include storing data in a properly secured location and anonymizing it. 

However, the criteria for doing so differ between the recommendations of biomedical ethics and the regulations proposed by certain anthropological associations. One example is whether or not interviews should be preserved. While biomedical research ethics committees recommend the destruction of such records after a specified period, some anthropological codes of ethics stipulate the researcher’s obligation to preserve the information for future studies.^24^ Unlike quantitative methods, which use large population samples, in qualitative research identification is much easier. The negative implications of unwanted identification can vary in nature and entail not only reflecting on whether to include information that could identify informants (in cases where they do not wish to be identified), but also on whether to reveal the study sites, and even on whether a research project is feasible without disclosing the group, institution, or context under study, an issue that is, in turn, linked to the type of research output (such as a book or a video).^2^

Now, what if the aim is precisely to produce evidence demonstrating the relationship between the practices of an individual, social group, or institution and the violation of other people’s human rights? Should we then defend the right to privacy and adhere to the principle of confidentiality? In social research, it is assumed that even when studying the social organization of harm and its protagonists, we must preserve the anonymity of the sources, as well as of the individuals to whom they refer, while making visible the social relations and contexts that foster and reproduce such vulnerability.^4^ Even in cases involving illegality, the transposition between police investigation and ethnographic research -when it entails breaching the principle of confidentiality- is generally unwelcome.^6^ In other words, if we request information in exchange for confidentiality, it is the social credibility in our ability to uphold this principle that enables us to continue working.

Cases in which confidentiality is not to be preserved relate to prior agreements made with the participant regarding the type of information provided, as well as to a court order linked to current legislation. Although it is uncommon for anthropologists to be legally required to reveal their sources, this could happen^15^ and, unlike medicine or law, we lack such explicit professional regulations. Various studies have documented the ethical dilemmas that arise in such situations, for example, when we witness acts of violence or receive confessions of future harm.^39^ Some remind us of the importance of situational ethics^18^^)^ and of the reactive strategies that emerge in the field,^40^ as well as of the need to carry out informed actions -often not easy to discern individually- that seek to assess the possible consequences of our practices, minimizing harm to the individuals and communities who share their intimacy with us, as well as to ourselves and to our profession. We will now examine how this becomes more complex in light of prevailing definitions and dominant uses of informed consent.

Informed consent seeks to ensure that the individuals with whom we conduct research are aware of the objectives, methods, risks (whether expected or unforeseen but possible), benefits (or lack thereof), social value, type of expected participation, and potential uses of the research, as well as the principles of voluntariness and the right to information and confidentiality, in order to decide whether or not to participate. Depending on ethical and disciplinary traditions and approaches, consent and assent (in the case of participants who are legally unable to provide the former) may be given orally, recorded in audio, or documented in a written consent or assent form. With some exceptions that justify it,^41^ it is recommended that consent be obtained at the outset of the research. While it is a fundamental principle to which any research should aspire, it is more useful to understand it as an ongoing process rather than as a completed act. In this sense, all research contains a concealed or incomplete component of informed consent,^4^ and there may even be cases in which not complying with informed consent is desirable, such as when working with individuals or collectives who, in contexts of impunity, violate human rights.^4^

An example of the complex interplay between ethnographic and police investigation, and between academic affiliation and activism, is Nancy Scheper-Hughes’s undercover research in the United States and South Africa on organ trafficking.^6^ While this reflects the specific characteristics of the research process, as well as the multiple affiliations involved, it is less common to discuss which concealed elements are present in our research, whether these are a deliberate methodological choice or an inevitable part of the process, and for which topics and populations such concealment should or should not be made explicit.^4^ There are three stages in which informed consent may be compromised: a) during access to the field and data collection; b) in the management of research results; and c) in the temporality of the research intent.^4^ The first occurs when negotiating objectives and strategies that change over the course of fieldwork, or when such objectives are partially disclosed to secure entry into the field, or provided only to gatekeepers and not to all actors involved. The same applies to the return of the final research product when it is not presented in an accessible language or format, is addressed only to certain sectors of the studied collectives, or contains only partial information. Temporality also plays a role, for example, when conducting autoethnographies that retrieve personal information involving third parties, initially produced without a research intent -and therefore without informed consent- and later turned into “publishable material”.^4^ This can also occur with the new uses of ethnographic material collected in the past. Strategies to address this include acknowledging that obtaining informed consent once is insufficient and that it must be negotiated successively, which also includes returning the final product.^2^

A concern identified within the Social Sciences is that informed consent is often understood from the perspective of an autonomous and rational subject who freely expresses their willingness to participate in research, without taking into account the constraints that may mediate such a decision.^41^ Given that our research frequently involves vulnerable populations with whom there are power asymmetries, the principle of voluntariness may be exercised in contexts of limited autonomy. This is the case, for instance, of users of a health service referred by staff who fear negative repercussions. The very format of the consent process may be perceived as reinforcing inequality within power relations.^42^

Some strategies to minimize the influence of power relations in obtaining informed consent include having it secured through individuals who are meaningful to the participant and different from the researcher,^2^ or sending it sufficiently in advance so that it can be reviewed. Other challenges arise from assuming the categories of “consent” and “information” as universal without adequate social and cultural adaptation,^(42^ from the bureaucratic and difficult-to-understand language used in consent forms,^42^ or from the mandatory signature of the document, which may put participants at risk when, for example, they are involved in illicit activities.^6^ It is also uncommon for all potential uses of the collected information to be made explicit, or for participants to always receive a copy of the informed consent.^27^

Current practices often seem more concerned with shielding institutions from potential legal claims than with the ethical value of informed consent or the commitment to relationships of trust, transparency, and reciprocity between researchers and participants.^42^ Undoubtedly, despite being the most debated process within the Social Sciences, it still requires a greater decentering from the biomedical field and the behavioral sciences focused on quantification, as well as a more visible and collective discussion from the Social Sciences and qualitative methodologies.

A similar issue arises with the validity of research results, which is generally conceived from a quantitative, positivist, and technical perspective that may obscure the ideological component in their evaluation and exclude or fail to recognize the characteristics of qualitative research. This will be discussed below.

## VALIDITY OF RESULTS

The validity of research results is related to Research Ethics insofar as it can affect their social value, the protection of participants, scientific credibility, or the responsible use of resources. While validity is a criterion shared by both quantitative and qualitative research -referring to whether the research can generate reliable and accurate evidence about what it aims to study- there are notable differences in how it is defined and used. One key difference is that quantitative research seeks objectivity and statistical generalization of its findings, whereas qualitative research, and ethnographic methods in particular, on the one hand, acknowledge that scientific data are social constructions involving the subjectivity of the researcher; and, on the other hand, may aspire to a typological or analytical generalization of findings.^43^

In this sense, quantitative research tends to minimize or control bias through statistical tools (standardization, probabilistic sampling, control groups, etc.), whereas qualitative research assumes that its primary task is to monitor, describe, and explain such bias. These differences have implications when a Research Ethics Committee whose members lack experience in qualitative research may fail to understand, for example, the value of participant observation for triangulating interview data; may consider that a case study with a sample of 20 people is numerically insufficient for result validity; or may determine that the “snowball” technique does not guarantee the right of all subjects to participate insofar as it directs participant selection.^44^

Another aspect of validity lies in the value attributed to evidence in different types of professional practice, such as in the interaction between anthropological expert testimony and the legal field, where anthropologists must adhere to ethical and scientific principles grounded in empirical evidence, which differ from those of lawyers, who are obligated to defend their client.^6^

Finally, another problematic dimension in the use of validity criteria has been identified when they serve to conceal the ideologies underlying the approval or rejection of socially sensitive protocols.^44^ For example, appeals to scientific objectivity have been used to withhold validation from research projects with explicitly political aims, such as the fight against Apartheid or homophobia, through inquiries into institutional discrimination.^44^^)^ In this regard, Research Ethics Committees do not operate in an ideological vacuum, and it is ethically questionable to hide behind scientific validity as a way to avoid openly discussing the ideologies,^44^ prejudices, and implicit assumptions of their members.

Moreover, it is not common for their implementation and functioning to be the subject of in-depth research,^45^ particularly in Global South contexts such as Latin America.^23^ This raises questions such as: Does the validity of results relate to the research method and design, or to their capacity to help address the problems faced by the communities with whom we work?^46^ How do we explain that validity, and for whom are our results valid -or not?

The following section will examine some of the scope and challenges of framing Research Ethics from the perspective of the communities -often subalternized- that participate in our studies.

## RESEARCH ETHICS FROM BELOW

Research ethics is not defined and evaluated solely by researchers; it is also shaped by the individuals and collectives who participate in our studies as a way of protecting themselves from us. Some communities, such as the Zapatistas in Chiapas, have considered that the potential risks outweigh the benefits -for instance, the use of published information by counterinsurgent state actors and/or organized crime- which has led them to restrict research in their territories.^4^

However, the deliberations that communities undertake to grant researchers access to the field are not always linked to research ethics, and, in the face of externally imposed criteria, various communities in the Global South have deemed it important to develop responses. One such response has been the creation of community-based Research Ethics Committees, which incorporate definitions and uses of ethics produced both by academia and by the community^27^^,^^47^ and, unlike other types of community groups, review research protocols from an ethical perspective, with specific training in research ethics and methods.^27^

Another way of co-producing “ethics from below” has been through the participation of representatives from the studied communities in Research Ethics Committees. Several international declarations highlight the importance of such participation; however, it does not always occur, nor is it always made explicit why their involvement is important, what their functions will be, or whether they are considered peers in the Committee’s decision-making process.^48^

Some identified challenges include multiple definitions of the concepts of community and representation, the lack of understanding regarding the importance of their role, and power imbalances in relation to the professionals who make up the Committees.^3^^,^^49^

Another existing “from below” approach is participatory ethics within research grounded in Participatory Action Research (PAR) or in approaches that, with distinct characteristics, stem from it, such as the “community-based participatory research” model, common in the health field. Due to its methodological and epistemological characteristics, Participatory Action Research includes reflections on research ethics that involve the participation of communities. This is evident in the joint involvement of researchers and community members in shaping research demands, in the research process itself, in the results, in the outputs, and in their uses, covering definitions and actions related to informed consent, risk/benefit/social value assessments, and the validity of data.

Some orientations within Participatory Action Research emphasize the need for epistemic justice through the recognition and valuing of the epistemologies, knowledge, theories, and experiences of participants, as well as their vindication in the face of the hegemony of both Global North ethics and theories^49^ and of Global South academia, which likewise reproduces inequalities. Nonetheless, several publications have pointed to the need for specific guidelines and conceptual frameworks for this type of research, as it is often assumed that inclusion, mutuality, benefit, and community control will be fulfilled, without specifying how this will be done or verified.^46^

A question that arises is whether basing research ethics on what the communities we work with define as ethical could be problematic, since what one community considers ethical, another may not.^50^ The same applies within a single community when we work, for example, with specific social groups who are marginalized by the community itself and who have limited or no capacity for collective mobilization within that community.

Another characteristic of Participatory Action Research is that it does not always fulfill all three of its components and often ends up being more research than action, or more action than participation or research. Likewise, participation is defined in widely varying ways, and in some cases, researchers’ definitions do not align with those of the studied communities,^51^ with ethical implications for the pre-established principles, which must be adapted, negotiated, and explained.

In response to the problems inherent in ethics designed from outside the communities, recent years have seen the development of recommendations on ethics and social participation (community engagement) -as one expression of Participatory Action Research- in diverse research contexts such as humanitarian aid,^52^ formulated in the form of ethical guidelines and competencies articulated through codes, manuals, and other formats.

If research ethics “from below” draws on diverse experiences such as those described here, what are we doing within our institutions to discuss and decide collectively on ethics from the perspective of the Social Sciences?

## INSTITUTIONAL EXPERIENCES IN MEXICO

For several years, I have been involved in the implementation and development of two institutional experiences evaluating ethical aspects of anthropological research at the *Centro de Investigaciones y Estudios Superiores en Antropología Social* (CIESAS) in Mexico. The first is a health-focused Research Ethics Committee (REC), which was proposed approximately ten years ago by Graciela Freyermuth, Paola Sesia, and myself, all affiliated with the institution. Its creation arose from the need for a collegial space for discussion and evaluation of research ethics in the health field, given the limitations we perceived in conducting such evaluations individually, and the growing demand from various institutions and scientific journals for formal ethical review of our protocols in order to conduct fieldwork, participate in inter-institutional research, or publish.

Another motivation was to avoid reliance on biomedical ethics committees, which often lack sensitivity to social research, especially in the absence of an REC within our institution. For the design and implementation of the CIESAS REC -which I coordinated from 2020 to 2024- a protocol was developed based on existing legislation, specifically the *Ley General de Salud*^26^ and the *Comisión Nacional de Bioética* (Combioética)^53^. The guidelines were adapted to anthropological research, taking into account several of the issues discussed here, while also carrying out the necessary steps for its institutionalization.

This process involved colleagues from CIESAS as well as from other Mexican institutions, such as the *Instituto Nacional de Salud Pública*. To ensure it did not take on a coercive character, the REC was established for researchers who voluntarily sought advice or a formal review of their protocols. Its characteristics are similar to other RECs in Mexico, with the distinction that it always includes experts in qualitative social research.

Although dedicated exclusively to the health field, it gradually began receiving requests from CIESAS researchers and external collaborators working in other areas, prompting reflection on its structure and scope. Despite its relative youth, the committee has carried out detailed evaluations of numerous protocols, providing feedback tailored to the qualitative nature of our research, aimed at ensuring adherence to social research ethics in health, and at the same time compiling materials to support the ongoing training of its members.

This is not the only REC in the Mexican academic sphere adapted to the Social Sciences; however, such committees remain far less numerous than those found in institutions dedicated to health research. As a form of self-critique, and recognizing that its first stage concentrated efforts on establishing the foundations for its functioning and formalization, I believe it would be advisable to: a) involve the social groups participating in the research in the evaluation process; b) raise awareness of the importance of the REC’s work to encourage broader academic community participation in its development; and c) create spaces for discussion of protocol evaluations with their authors, moving beyond the current format of email and document exchanges.

The second experience in which I have participated is the Risk Committee, established in 2020 and currently active, as an ad-hoc committee of the Academic Council of the Graduate Program in Anthropology at CIESAS, Mexico City. This committee has been coordinated by Susann Vallentine Hjorn, with implementation and development contributions from Hiroko Asakura and Carolina Robledo Silvestre, all three researchers affiliated with CIESAS. Its primary objective is to provide recommendations based on risk assessments for the fieldwork of graduate students.

With the onset of COVID-19, I was invited to participate in order to contribute to the evaluation of risk and the mitigation of virus transmission. While the Risk Committee was not conceived as a Research Ethics Committee, many of its recommendations are closely linked to social research ethics. Its consultative nature has been important in guiding both individual and collective recommendations, which include participation in risk workshops and the analysis and follow-up of incidents occurring after fieldwork, on topics such as exposure to violence or health risks.

In the field of ethics, and specifically regarding COVID-19, the Risk Committee has not only recommended strategies to mitigate risk for students, research participants, and their families and contacts, but has also discouraged research projects where potential harm would outweigh benefits, suggesting virtual or remote modalities in contexts of voluntary confinement.

Although these experiences do not constitute solutions that always resolve all research ethics dilemmas in every context, nor do they guarantee the ethical behavior of individuals, they do represent a collective accompaniment that seeks to foster constructive critique, research autonomy, and, at the same time, monitor power relations among those involved in a study and the communities to which they belong, as well as their potential harm.

In the following section, we propose a series of recommendations based on the experiences and publications discussed in this text, aimed at social scientists and the communities with which they work, at Research Ethics Committees and the institutions in which they operate, and at scientific journals.

### Recommendations for Social Research Ethics with Fewer Gaps and Greater Possibilities

#### For Social Scientists and the Communities with Which They Work

- Promote, within institutions, spaces for collective discussion on research ethics that include students, researchers, and members of the communities under study. These spaces would allow for the sharing of experiences and materials to be reviewed and discussed, with the aim of improving individual and collective deliberations in our research. From these discussions, concrete recommendations should be developed in accessible formats.
- Encourage collegial committees, with a consultative function, to conduct formal evaluations of research protocols. These committees should be adapted to the characteristics of both qualitative and quantitative research in the Social Sciences, with criteria that allow discussion in relation to existing guidelines, and should operate transparently, on a rotating basis, and involve voluntary participation from the academic community, either as evaluators or as subjects of evaluation.
- Recognize research participants as individuals with rights and as members of communities with collective rights, whose knowledge can contribute to ethical practice and to the recommendations of Research Ethics Committees.
- Conduct qualitative research on the functioning of Research Ethics Committees within health institutions, universities, companies, international organizations, or public ministries to understand the scope and limitations of these resources and to invite discussion.
- Research teams should prioritize publishing their results in journals that promote free and open access to published content, thereby enabling access for the general population and especially for communities that face greater socioeconomic and digital disadvantages in accessing scientific information.
- I recommend that, despite the above, members of the studied communities establish their own Research Ethics Committees to evaluate our research and protect themselves from potential risks, generating guidelines with ethical competencies (and legal resources to defend them) in manuals or regulations that can be shared and discussed with other communities.

#### For Research Ethics Committees and the Institutions in Which They Operate

- Consider that research ethics is not limited to the operation of a REC to approve research, nor to obtaining informed consent for data collection, but rather constitutes a process of continuous reflection and discussion that spans the entire research project, from conception to outputs.
- Include experts in qualitative social research methods.
- Promote the participation of representatives from the studied communities in RECs, commissions, or other collegial spaces.
- When not evident in the evaluated research protocol, pose questions to researchers that address *for what purpose* and *for whom*, and not only *how*, *what*, *when*, or *who*.
- Promote clear principles, training, and guidelines regarding sensitive issues related to vulnerable populations and human rights, as well as the diverse types of research methods and existing epistemological traditions.
- Include specialists with expertise in community ethics, participatory action research, and social participation, and, where possible, with experience in the contexts where the research will be conducted.
- Foster spaces for constructive discussion on ethics with research teams, so that evaluations are neither a mere bureaucratic process nor an authoritarian exercise.
- Ensure that RECs have the resources to carry out their work continuously and to follow up on research whose protocols have been evaluated.
- Examine whether research protocols are informed by values and ideologies (mediated by the researchers, their institutions, and/or funding agencies) that could harm participating communities, and propose strategies to address these issues.
- Engage in collective reflection on the influence of the ideologies, values, and implicit assumptions of REC members (and their affiliated communities and institutions) on the definition of what is ethical, as well as on the role these processes play and their contradictions in the functioning of the REC.
- Implement effective mechanisms to prevent RECs from becoming spaces of power where conflicts of interest or personal agendas are concealed while external ones are highlighted.
- Propose integrated discussions within RECs, or within other specialized committees, to analyze the ethics of research funding agencies and institutions with respect to their agendas and the potential uses of research outputs; this includes addressing potential conflicts of interest of both researchers and funding agencies.

#### For Scientific Journals

- Encourage that submitted articles provide approval from a Research Ethics Committee (REC) or an analogous body. If the authors’ institution does not have one, or if the authors chose not to submit the protocol, request information on the reasons it was not evaluated and on the measures taken to ensure the research adhered to necessary ethical standards. Provide an additional space, up to the maximum allowed length, for authors to describe this.
- If necessary, request supplementary materials to verify that the research complied with required ethical criteria, such as informed consent forms or additional information on data protection measures.
- Include experts in social research ethics on editorial boards who can advise the editorial team.
- Promote editorial spaces for discussion on research ethics, including universal principles, local specificities, the power dynamics that determine what is considered ethical, the implications and characteristics in each discipline, and normative or alternative practices that may be effective.
- Critically review whether the journal’s publications have perpetuated classism, racism, sexism, xenophobia, homophobia, ableism, or any other form of oppression, as well as extractive or dispossessive practices affecting ecosystems, regions, or knowledge systems. If so, implement reparative measures by promoting publication spaces and circuits that make the harm visible, amplify the voices of those affected, and outline ways to address it.

## FINAL CONSIDERATIONS

This article has discussed some of the absences, urgencies, and possibilities of research ethics in social research in Latin America, with various examples related to anthropology and health. Three axes of analysis have been proposed: the limited collective and institutionalized discussions and their negative impact on professional practice; the exclusion of the specificities of social research and the analysis of power relations due to the imposition of health sciences from the Global North; and the existence of certain practices within academic institutions and studied communities that have sought to address these issues.

In this sense, research ethics is always political insofar as it should reflect on unequal power relations and their consequences regarding the definition of what is ethical, the choice of what is studied, how it is studied, and for what purpose, acting collectively on the possibilities for transformation as the basis of social value, risk/benefit evaluation, informed consent, confidentiality, and the validity of results. To achieve this, it is essential to cultivate the diverse terrain of ethics of care^54^^)^ in contrast to the monoculture of productivity ethics.^55^
